# Investigation of Device- and Circuit-Level Reliability of Inverse-Mode Silicon-Germanium Heterojunction Bipolar Transistors

**DOI:** 10.3390/s24227130

**Published:** 2024-11-06

**Authors:** Taeyeong Kim, Garam Kim, Moon-Kyu Cho, John D. Cressler, Jaeduk Han, Ickhyun Song

**Affiliations:** 1 Department of Artificial Intelligence Semiconductor Engineering, Hanyang University, Seoul 04763, Republic of Korea; kd97050@hanyang.ac.kr; 2 Department of Electronic Engineering, Myongji University, Yongin-si 17058, Gyeonggi-do, Republic of Korea; garamkim@mju.ac.kr; 3 Department of Computer Engineering, Korea National University of Transportation, Chungju-si 27469, Chungcheongbuk-do, Republic of Korea; moonkyu.cho@ut.ac.kr; 4 School of Electrical and Computer Engineering, Georgia Institute of Technology, Atlanta, GA 30318, USA; cressler@ece.gatech.edu; 5 Department of Electronic Engineering, Hanyang University, Seoul 04763, Republic of Korea

**Keywords:** avalanche, breakdown, circuit-level reliability, electrical stress, forward mode (FM), Gummel, hot carrier, heterojunction bipolar transistor (HBT), inverse mode (IM), silicon germanium (SiGe), small-signal model

## Abstract

The reliability of inverse-mode silicon-germanium (SiGe) heterojunction bipolar transistors (HBTs) under dc stress and its potential impact on the performance of basic analog amplifiers are investigated. In order to properly reflect the stress effects in various circuit applications, the degradations under three different configurations (active bias, diode connection, and off state) were experimentally characterized with the stress voltages applied up to 3000 s for each case. Based on the changes in the Gummel response, the degradations in device parameters such as current gain (β), transconductance (g_m_), and base-to-emitter resistance (r_π_) were extracted and compared with the forward-mode counterpart. In addition, with the use of a small-signal equivalent model of a SiGe HBT, simple single-stage analog amplifiers were simulated as representative examples and their circuit-level performance metrics including gain and bandwidth were studied to estimate degradation characteristics with accumulated stress. It was found that transimpedance gain decreases and operation bandwidth increases to different levels due to device degradation, whereas a voltage amplifier exhibited much less changes.

## 1. Introduction

Silicon-germanium (SiGe) heterojunction bipolar transistors (HBTs) have provided unique advantages over conventional CMOS technologies in terms of high-frequency operation, noise characteristics, and large-signal performance, supporting seamless integration with CMOS technology [1,2,3]. In addition, a SiGe HBT is known to be robust against the total ionizing dose (TID) up to multi-Mrad irradiation by virtue of the intrinsic structure of the device that is less dependent on the oxide quality [4,5]. Regarding single-event effects (SEEs), which happen when a high-energy particle hits an active device and generates many excess charge carriers, however, SiGe technology has been known to be susceptible due to its junction-based operation [6,7,8,9,10,11]. Therefore, the use of a SiGe HBT under an SEE-intense environment (e.g., deep space) may impose a serious issue in signal integrity. Among various remedies and solutions, the use of an inverse-mode (IM) operation, which utilizes potential barriers and a low electric field, has been proposed as a viable radiation-hardening-by-design technique [12].

In most analog applications, forward mode (FM) SiGe HBTs are used to maximize circuit performance metrics. Unlike FM SiGe HBTs, the IM operation, where the physical emitter has higher voltage potential than the collector, is effective in mitigating the impact of single-event transients (SETs) and associated signal distortions for extreme-environment applications. This is due to the reduced transient peaks and/or durations compared with FM operation [12,13,14,15]. Since the IM configuration does not alter device intrinsic structure, there is no need to modify mask layers or include other peripheral circuitry [14,15]. On the other hand, one of the major concerns of using IM SiGe HBTs includes degraded performance due to unfavorable device dimensions and the doping profile [15,16,17]. Fortunately, with the help of technology scaling, dc and ac performance parameters have been improved for IM SiGe HBTs (e.g., current gain > 100, and unity-gain frequency > 50 GHz) as well as FM counterparts [18,19].

When it comes to device-level reliability under electrical stress, however, there have been few studies about degradations associated with IM operation [20,21]. Since most papers have focused on FM SiGe HBTs in the literature [2,3,4,5,6,7,8,9,10,11,12,13,14,15,20,21,22,23,24], proper design guidelines or performance estimation associated with IM SiGe HBTs is more or less limited. Whereas some characteristics of IM SiGe HBTs might be inferred from reliability results of FM cases, in order to fully exploit the benefits and compensate for the risks of IM SiGe HBTs, it is critical to analyze their degradation characteristics under electrical stress over time and evaluate robustness or weaknesses. In addition, the degradation results of a device are more beneficial if they are related to circuit-level stress conditions. As most SiGe HBTs are employed in analog circuit applications, parameter changes in a device affect circuit performance significantly. Hence, a relevant correlation between an IM SiGe HBT and a circuit in comparison with FM operation needs to be investigated.

In this paper, we study the different trends of degradations under three electrical stress conditions for a SiGe HBT in an amplifier. Three distinct stress conditions are active-bias, diode-connection, and off-state configurations, all of which are widely used in many analog circuits. The findings of this work can be used for the design and analysis of robust reliable circuits and systems and the prediction of performance. The organization of the paper is as follows. In Section 2, the stress conditions are described in detail and in Section 3, degradation results will be presented and analyzed. Section 4 will discuss expected circuit-level performance degradations based on the device characteristics and Section 5 summarizes the findings of this work.

## 2. Hardware Preparation and Test Setup

The devices under test (DUTs) used in this work were all NPN SiGe HBTs. They were fabricated in GlobalFoundries’ 130 nm SiGe BiCMOS technology platform (8HP), which provides the unity-gain frequency (f_T_) of 200 GHz and the maximum oscillation frequency (f_MAX_) of 265 GHz [25]. Among available transistor options in the process, the high-performance version of SiGe HBTs was chosen. The (physical) emitter area was 2.5 μm (length) × 0.12 μm (width). In addition, the terminal organization of DUTs was configured as the C-B-E-B-C layout (C: collector, B: base, E: emitter), in which the collector current is distributed into two separate paths from the center (emitter) to both ends (collector).

As a versatile electronic component, a SiGe HBT is utilized in a different configurations in a variety of analog circuits. It can be employed as a voltage/current gain element, a biasing component, and a controlling device. Based on its key usage in circuit applications, it is exposed to the following stress conditions: (1) active bias, (2) diode connection, and (3) off state. Under each condition, the characteristics of a SiGe HBT were measured and compared for both the forward mode (FM) and inverse mode (IM). All devices were measured in an on-wafer test setup using a probe station and Keysight 4155C. The focus of this work is on single HBTs that are biased to operate in FM and IM each, under three distinct stress conditions.

**Figure 1:**
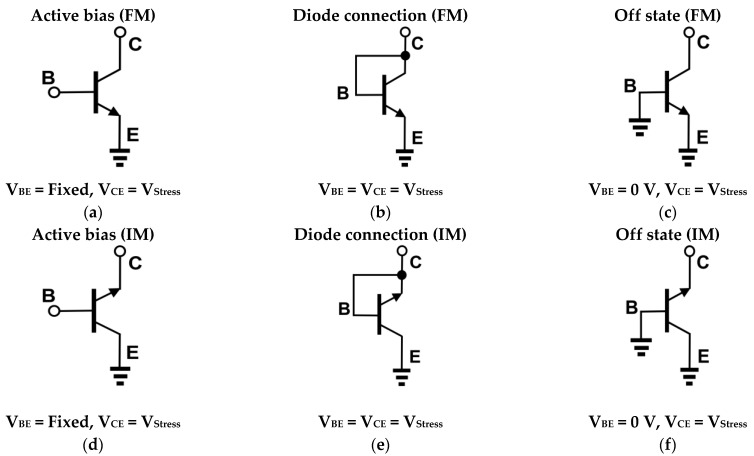
Stress conditions of a SiGe HBT in forward mode (**a**–**c**), and inverse mode (IM) (**d**–**f**). For an IM SiGe HBT, terminal names of C and E denote an electrical collector and an electrical emitter, respectively.

In Figure 1, stress conditions including terminal connection and DC voltages are shown. The left column (Figure 1a–c) represents FM stress cases, whereas the right column (Figure 1d–f) is for IM cases. After each electrical stress, device Gummel was measured under V_CB_ = 0 V. In IM configurations, the electrical connections of the physical collector and the emitter were swapped electrically. Under the active-bias stress conditions (see Figure 1a,d), the collector and the emitter terminals were reverse-biased and the base current was applied such that at the collector, the current was about 1 mA, setting the base-to-emitter voltage (V_BE_) accordingly. Next, in the diode-connection cases (Figure 1b,e), the base and the electrical collector terminals are tied together and a high V_CE_ voltage was applied to stress a DUT. Lastly, Figure 1c,f illustrate the off-state stress condition, where the base is connected to a ground node. For each setup, the stress time was set to 3000 s, at which degradations were noticeable for all combinations of the operation mode and stress configurations. The stress voltages were set by finding the maximum voltage under which devices survived for both FM and IM. For the active-bias and off-state conditions, V_CE_ was swept with a 0.1 V step, whereas in the diode-connection condition, V_CE_ (=V_BE_) was swept with a 0.04 V step. From the measured Gummel, degradation characteristics of devices were compared and major device parameters such as current gain and transconductance were extracted for a performance analysis.

## 3. Experimental Results

**Figure 2:**
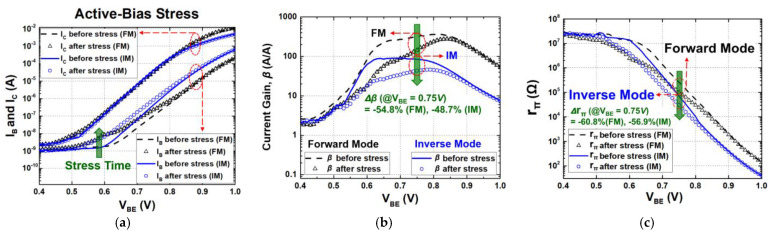
(**a**) Gummel plot (I_C_ and I_B_ versus V_BE_); (**b**) degradation of current gain (*β*); (**c**) base-to-emitter resistance (*r_π_*) degradation under active-bias stress condition.

**Figure 3:**
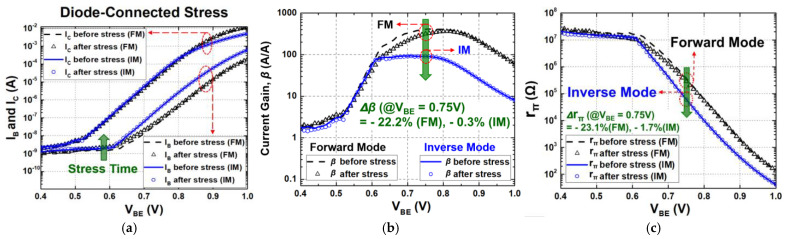
(**a**) Gummel plot (I_C_ and I_B_ versus V_BE_); (**b**) degradation of current gain (*β*); (**c**) base-to-emitter resistance (*r_π_*) degradation under diode-connection stress condition.

**Figure 4:**
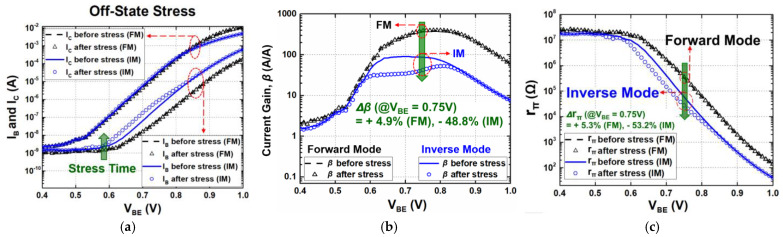
(**a**) Gummel plot (I_C_ and I_B_ versus V_BE_); (**b**) degradation of current gain (*β*); (**c**) base-to-emitter resistance (*r_π_*) degradation under off-state stress condition.

The overall transistor characteristics under each stress condition (active bias, diode connection, and off state) are shown in Figure 2, Figure 3 and Figure 4, respectively. The degradations in the electrical collector current (I_C_) and the base current (I_B_) of a SiGe HBT were measured for both FM and IM before and after 3000 s of stress time. As shown in Figure 2a, Figure 3a and Figure 4a, the changes in I_C_ were much less than those of I_B_ in general. Because of an increase in I_B_, current gain (β) reduces over stress and the degradations are severe when V_BE_ is below approximately 0.8 V, whereas moderate or negligible deviations were observed with high V_BE_ [3,26,27].

To understand the degradation mechanism of SiGe HBTs by electrical stress, it is necessary to investigate hot carriers and the avalanche effect. The formation of hot carriers in the CB depletion of SiGe HBTs is triggered by the high reverse bias CB voltage (V_CB_), which generates a high electric field and results in the generation of high-energy hot carriers within the emitter–base (EB) spacer [26,27,28,29]. These minority carriers at the base shift toward the collector–base (CB) depletion region due to the high CB voltage (and electric field). When a hot carrier with sufficient energy reaches the oxide, it can form a trap at the emitter–base (EB) spacer and shallow trench isolation (STI) oxide interface through impact ionization and cause the avalanche effect [26,27,28,29,30]. In the literature, device breakdown is characterized with the collector–emitter breakdown voltage with an open base (BV_CEO_) and the base–collector breakdown voltage with an open emitter (BV_CBO_), which are commonly used to determine the operating limits of SiGe HBTs [26,30,31,32,33]. For example, BV_CEO_ can be obtained by measuring the I_C_ when I_B_ is zero and is the point at which base current reversal (BCR) begins during the forward-active operation. Therefore, if V_CE_ is greater than BV_CEO_, device breakdown is triggered by multiple carriers, which leads to a significant increase in current via positive feedback [34].

An increase in the base current of a SiGe HBT by sufficient V_CE_ can be modeled with the avalanche effect and it can be expressed as follows [35].

(1) $$I_{B}=\frac{I_{C,0}}{\beta_{0}}\times e^{\frac{V_{be}}{V_{T}}}-(M-1)I_{C,0}\times e^{\frac{V_{be}}{V_{T}}}$$

In (1), I_C,0_ is the collector current before stress, V_T_ is the thermal voltage, β_0_ is the DC current gain, and M is the avalanche-current multiplication factor. With a fitting parameter n, M is written below.

(2) $$M=\frac{1}{1-{\left(\frac{V_{CB}}{{BV}_{CBO}}\right)}^{n}}$$

BV_CBO_ is typically a few times larger than BV_CEO_ and BV_CEO_ can be written as follows [35].

(3) $${BV}_{CEO}=V_{BE}+\frac{{BV}_{CBO}}{\sqrt[{n}]{\beta_{0}+1}}$$

Based on the above equations, degradations in the base current due to the avalanche effects can be further analyzed along with physics-based device simulations.

The non-ideal base current increases with the stress voltage. Consequently, the current gain is reduced by the leakage current at the base, which eventually degrades the circuit/system performance [36,37,38]. Whereas the key mechanism of degradation is related to hot carriers in a SiGe HBT, however, the electrical configuration of a device in a circuit leads to different performance changes. In a variety of analog applications, a SiGe HBT is under and among an active bias, diode connection, and off state, which will not present the same degradation characteristics between FM and IM. Therefore, for circuits with IM SiGe HBTs, it is pivotal to understand device characteristics under each stress condition for predicting long-term reliability issues in the design phase and dealing with potential performance loss.

In the active-bias configuration, the stress voltage was applied by keeping the electrical EB junction forward-biased and the electrical CB junction reverse-biased (Figure 1a,d). Here, the applied stress (V_CE_) was 2.7 V, and the total stress time was 3000 s. As shown in Figure 2a, I_B_ increased due to trap generation as the stress was accumulated, whereas the changes in I_C_ were much less. Similar degradation trends were observed in both FM and IM operation, but FM was more susceptible to the stress. This is because unlike FM, the STI oxide in IM has fewer mid-gap states, so the increase in the base current is lower [15,16,17]. Moreover, the STI oxide interface is located further away from the EB depletion region at the neutral base [15,16]. In addition, the STI oxide in IM is already highly defective, which helps to limit the increase in the base current. The peak I_B_ increase was 121% and 95.1% with an electrical V_BE_ of 0.75 V in FM and IM, respectively.

Regarding the device performance of a SiGe HBT, current gain (β) versus V_BE_ before and after the active-bias stress is presented in Figure 2b. As implied from Figure 2b, β started to degrade when V_BE_ is below about 0.9 V. The largest reductions were observed when V_BE_ was at about 0.6–0.7 V and comparable degradations occurred in both FM and IM. These characteristics show that SiGe HBTs may suffer from potential performance loss under the active-bias stress if they operate in low-power applications. In these applications, typical V_BE_ ranges will be at around 0.75 V or less to provide moderate gain and low bias currents [19,31,39]. Specifically, under this bias voltage, the current gain was degraded by 54.8% and 48.7% in FM and IM operations, respectively, limiting the lifetime of the devices. In the aspect of device modeling, the base-to-emitter resistance (r_π_) versus V_BE_ is shown in Figure 2c. Like current gain in Figure 2b, r_π_ exhibits more degradation in the lower-V_BE_ region (V_BE_ < 0.7). After 3000 s of stress, r_π_ was reduced by 60.8% and 56.9% for FM and IM, respectively. These degraded resistances along with transconductance (g_m_) will be used in the small-signal model to predict circuit performance over stress in the next section. In summary, the degradations in device characteristics are severe in both FM and IM, but the degree of changes is less in IM than those in FM. With negligible degradations in I_C_, the increase in I_B_ is lower by 25.9% in IM than in FM, and consequently, β and r_π_ degrade by 6.1% and 3.9% less in IM, respectively.

In the case of diode-connection stress, the base and the collector terminals were tied together as a diode-connected device, and then, the same stress voltage was applied (Figure 1b,e). The base and the collector currents were measured for the stress time up to 3000 s and the applied stress voltages of V_BE_ (=V_CE_) were set to 1.22 V. In Figure 3a, similar to the active-bias condition, I_B_ increases as the stress accumulates, indicating that the stress voltage causes interface traps to form at the EB spacer oxide and STI edges [13,14,15,26,27,28,29]. Whereas the overall degradation was reduced in comparison with the active-bias stress, it is shown that there are relatively large variations in FM and little variations in IM. The former and the latter exhibit an increase in I_B_ by 30.13% and 1.73% in FM and IM, respectively, under the bias point with V_BE_ of 0.75 V. Since in diode-connection configuration, electrical base and collector terminals have the same voltage, the number of generated hot carriers is reduced due to the low avalanche effect, causing a small number of traps in the oxide layer. On the other hand, in IM, the STI region is less affected than the spacer in FM, leading to the better response in IM. After applying the stress voltage for 3000 s, the degradation of β is shown in Figure 3b. β decreases by 22.2% and 0.3% in FM and IM, respectively. And r_π_ shows a degradation of 23.1% and 1.7% in FM and IM, respectively (Figure 3c). Whereas the overall degradation for FM was less than the active-bias case, however, the reduction in β exceeds 10%, which may limit the lifetime of a given device. For IM, however, the decrease was 0.3% and 1.7%, respectively, which implies that an IM SiGe HBT can remain within a usable range of the lifetime in terms of long-term reliability [30].

In the off-state stress configuration, the base terminal was grounded, while the stress voltage is applied only to the electrical collector (Figure 1c,f). Stress time was the same as in the previous cases and the applied stress voltages were set to turn off the device; V_BE_ and V_CE_ were 0 V and 3.6 V, respectively. In contrast to the other stress conditions, it shows a little degradation in I_B_ in FM, but a significant increase in IM (Figure 4a). An increase of −4.9% and 113.2% was observed in FM and IM with a V_BE_ of 0.75 V, respectively. The small degradation in FM implies that the EB spacer is not introduced to additional traps. On the other hand, in IM, a high electric field applied across the EB junction leads to damages, and consequently, a leakage current due to breakdown effects. Figure 4b shows β versus V_BE_ before and after stress. It reads a 4.9% increase in FM and a 51.2% decrease in IM, which can be implied from the Gummel response. As shown in Figure 4c, r_π_ increases by 5.3% in FM, but decreases by 53.2% in IM after 3000 s of voltage stress. Since there were little changes in I_B_ under FM, the resulting decrease in r_π_ and β was negligibly small. It showed that a variation in current gain was 4.9% under the bias of V_BE_ = 750 mV after 3000 s of stress, which is within the boundary of 10% reduction in terms of device lifetime. In contrast to the previous active-bias and diode-connection conditions, the off state showed a worse degradation in IM. This shows that the off state is significantly affected by electrical stress in IM.

**Figure 5:**
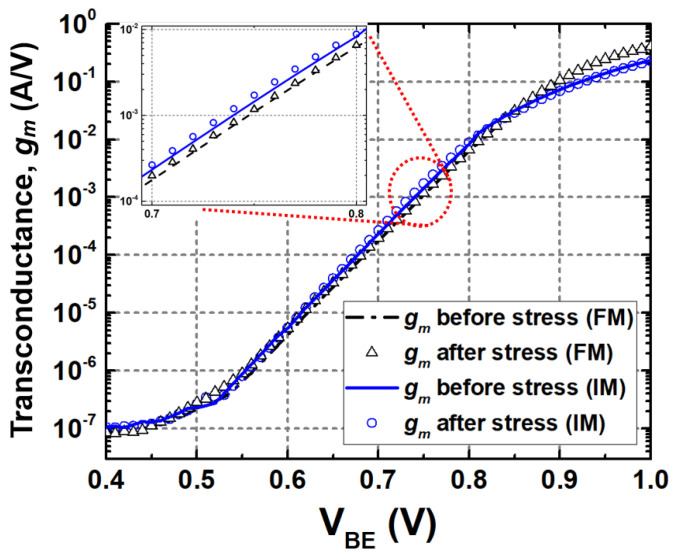
Transconductance (g_m_) before and after stress under active-bias condition in FM and IM.

Figure 5 shows g_m_ vs. V_BE_ of SiGe HBTs in FM and IM. For all stress conditions, degradations in I_C_ between the fresh and the stressed cases were much less than those of I_B_. Since g_m_ is, by definition, a partial derivative of I_C_ with respect to V_BE_, it will present as almost similar g_m_ as long as I_C_ does not change much. From the stress experiment, all three stress cases of FM and the diode-connection and off-state conditions of IM exhibited little deviations in g_m_ (less than about 7%) from the fresh states, whereas the active-bias condition of IM showed a slight increase by about 15%, depending on the bias voltage. For simplicity, a full-scale and a zoomed-in response of g_m_ under the active-bias condition only is plotted in Figure 5. Despite the fact that these deviations seem small, they can directly affect circuit performance numbers especially in a voltage-driven amplifier. More details will be discussed in Section 4.

**Figure 6:**
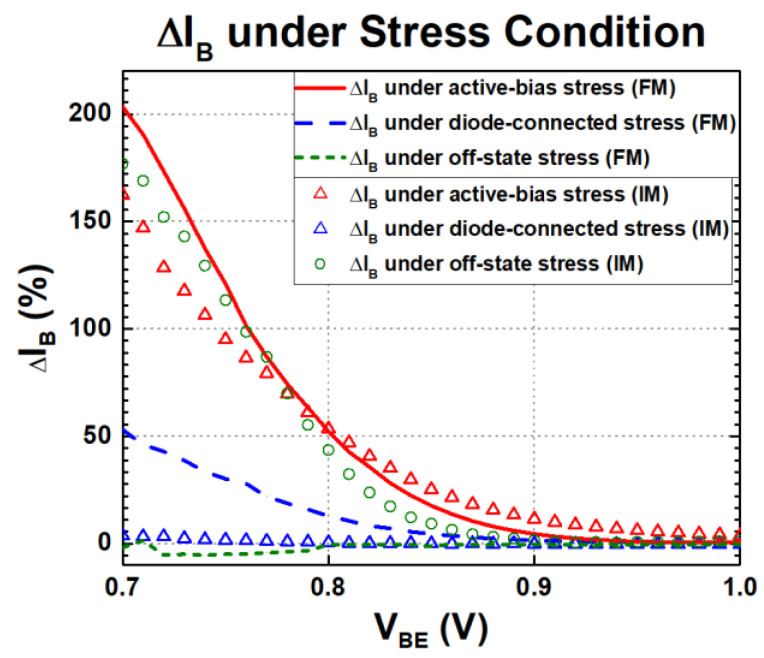
Changes in I_B_ under three stress conditions of FM and IM SiGe HBTs before and after stress.

Based on the degradation experiments, it is concluded that electrical stress leads to an increase in I_B_ of a SiGe HBT in general, thereby affecting device parameters such as β and r_π_. Depending on the stress configurations (active bias, diode connection, and off state) and operation mode (FM or IM), the degradation characteristics are different. From the device structure, the EB spacer and STI oxide are the main components that receive damages such as the generation of traps and mid-gap states. Increased reverse bias at the electrical CB junction causes impact ionization, which leads to the formation of energy carriers that can migrate to the EB spacer and STI oxide interface. As a result, these high-energy carriers create traps that lead to base current degradation, damaging the interface and shortening the device lifetime. The generation rates of hot electrons and hot holes at the STI interface are typically much higher than at the EB spacer interface. This results in higher trap density along the STI, indicating that more severe degradation is caused for FM than IM under stress, as shown in the Gummel characteristics before and after stress in Figure 6 [13,14,15,16,17,18,19]. The highly doped emitter (electric collector) in IM modulates the neutral base width more than FM, so there is less of an avalanche effect to form hot carriers. Therefore, there is a reduced number of hot carriers generated at the electrical CB junction in IM. With a lesser number of traps, the leakage current from the base is reduced, as shown in the Gummel characteristics of FM and IM under active-bias and diode-connection stress conditions in Figure 6 [15,40,41,42]. In contrast to active-bias and diode-connection stress conditions, off-state stress conditions show greater degradation in IM. This can be expected due to the higher doping concentration of the electrical collector in the presence of high collector–base voltage, which leads to a breakdown effect that does not occur in FM, resulting in the appearance of a leakage current at the base.

**Figure 7:**
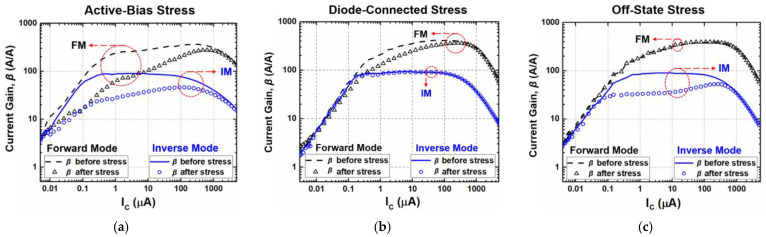
Degradations in current gain (*β*) versus I_C_ under (**a**) active-bias, (**b**) diode-connected, and (**c**) off-state stress conditions.

The overall device characteristics under three stress conditions in FM are summarized in Table 1. Three important parameters of a SiGe HBT including current gain, transconductance, and base-to-emitter resistance show different degradation results. Under the active-bias case, reductions were the most severe, showing a degradation in β more than 50%. It implies that normal operation as an amplifier would be significantly affected in terms of performance. On the other hand, the off-state configuration resulted in the least degradation. Thus, it is a good approach to ground the base terminal of a SiGe-HBT amplifier to minimize or avoid stress while it is not in operation. Table 2 shows the device characteristics under the three stress conditions in IM. The degradation characteristics are better in IM than FM in the active-bias and diode-connection configurations. Unlike these two states, in the off state, we can see that the degradation is greater in the IM than in the FM under electrical stress conditions, especially in the case of *β*, where the degradation characteristic is 51.2% in IM compared to 4.9% in FM, which is outside the degradation limit of 10% in IM. Therefore, it may be a good alternative to avoid the off-state configuration when using SiGe-HBT amplifiers as IM. Lastly, it is worth presenting the device degradations versus the collector current because bipolar transistors and circuits are fundamentally biased via I_C_. Figure 7 shows *β* versus I_C_ for the three stress states; Figure 7a, Figure 7b and Figure 7c illustrate the degradation characteristics of *β* under active-bias stress, diode-connected stress, and off-state stress conditions, respectively.

## 4. Simulation of Circuit-Level Degradation

**Figure 8:**
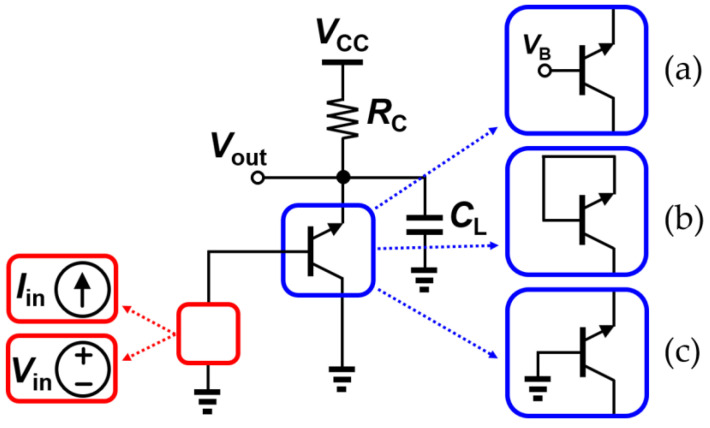
Basic schematic of TIA or voltage amplifier under (**a**) active-bias; (**b**) diode-connection; and (**c**) off-state conditions. Red boxes show types of signal sources and blue boxes show possible types of stress.

In order to study the aging effect of SiGe HBTs in a circuit, a SiGe-HBT-based transimpedance amplifier (TIA), which accepts a current input and generates a voltage output, and a SiGe-HBT voltage amplifier, which accepts a voltage input and generates a voltage output, were designed. The performances of amplifiers were evaluated with the small-signal models and process-design-kit models. On the left side of Figure 8, the upper and lower red boxes denote TIA and voltage amplifier configurations, respectively, whereas on the right side, the blue boxes show potential stress conditions. For normal operation, it is biased in the safe operation area (SOA) with a power supply (V_CC_) of 1.8 V and a load resistance of 20 kΩ. On the other hand, when under stress situations, V_CC_ is assumed to be raised up to 3.6 V.

In this situation, the transistors are under the same stress conditions as in the previous section. Figure 8a represents a typical common emitter (CE) amplifier that undergoes large V_CB_ stress, whereas Figure 8b shows that diode-connection stress is biased in a way such that V_BE_ and V_CE_ are matched under a 3.6 V power supply. Lastly, in the off-state stress condition, the based terminal is grounded, resulting in no I_C_ and having V_CE_ the same as the stress voltage, as shown in Figure 8c. Using the degraded small-signal parameters from different stress conditions, as investigated in Section 3, the small-signal performance under normal operation was re-simulated and compared with the initial performance. The device parameters used in the circuit are given in Table 3 and Table 4. They show the initial and degraded values of *g_m_*, *r_π_*, and the early voltage (*V_A_*) with stress, which was used to investigate the effect of stress on the circuit performance. The early effect (or the base-width modulation) of each device was included in the performance analysis. In addition, a load capacitance of 100 fF attached in parallel at the output node to represent a potential input impedance of a subsequent stage. For simple AC modeling, the base-to-emitter, base-to-collector (C_BC_), and collector-to-emitter (C_CE_) capacitances are extracted from the design kit model. Since it has been reported in the literature that the variations in device capacitance due to DC stress are almost negligible (less than 5%) [31,43], constant values were assumed in the following simulations.

**Figure 9:**
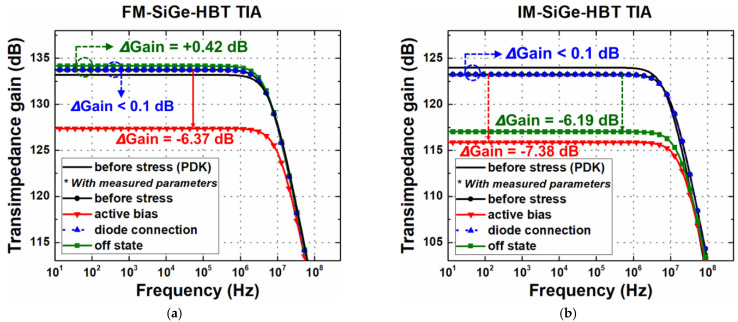
Bode plot of TIA gain under three stress conditions of (**a**) FM; and (**b**) IM (after 3000 s of stress).

The Bode plot of each stress condition in FM is shown in Figure 9a along with the pre-stress response. For a verification purpose, the results from the PDK models were simulated with Cadence Virtuoso [44] for a pre-stress condition and the overall differences were within only 1~2 dB. As discussed in Section 3, the stress-induced variations in base-to-emitter resistance (*r_π_*) and transconductance (*g_m_*) have different degradation characteristics for each state. In the active-bias stress, there were little variations in *g_m_* but a large degradation in *r_π_*, which implies that the gain will vary because the voltage applied between the base and the emitter (or simply, across *r_π_*) will be reduced. The overall degradation in TIA gain was the worst among three cases, exhibiting a reduction of about 6.4 dB. With regard to amplifier bandwidth, it is shown that the location of the dominant pole (at the output) is shifted toward a higher frequency, slightly increasing the bandwidth. This is due to the presence of the internal feedback capacitor C_BC_.

In contrast, the other two stress cases did not show significant changes in TIA gain and bandwidth. Regarding the diode-connection stress case, one thing to note is that when the V_CC_ is raised to 3.6 V, the effective bias applied at the base and collector node is about 0.8 V, which indicates that the device is still in the safe operation area (SOA). From the device test, it was verified that there was no noticeable degradation under this bias condition. Therefore, in the amplifier configuration, the performance variations are negligible. Lastly, in the off-state stress, the degradations in *r_π_* and *g_m_* result in a gain increase of 0.4 dB, which is attributed to a small increase in *r_π_* (see Figure 9a). In Figure 9b, before- and after-stress results in the IM TIA are shown. Similarly to the FM TIA case, it exhibits large degradation in the active-bias stress conditions and negligible changes in the diode connection in terms of gain and bandwidth. For the off-state stress, however, performance degradations were severe and close to those of the active-bias case, meaning that the IM operation may present long-term reliability risk, as implied from Table 2.

**Figure 10:**
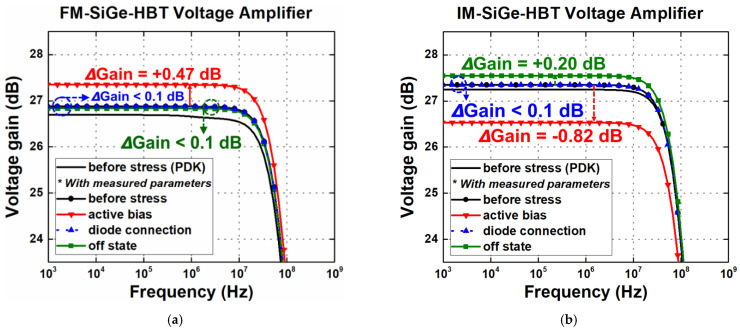
Bode plot of voltage amplifier gain under three stress conditions of (**a**) FM; and (**b**) IM (after 3000 s of stress).

In Figure 10, the Bode plots of the FM and IM SiGe-HBT voltage amplifiers are shown. In this amplifier configuration, the gain of the circuit is now directly dependent on *g_m_* and is not affected by the variations in *r_π_*, presenting similar trends to the characteristics of *g_m_* under the three stress conditions extracted in Section 3. The FM voltage amplifier showed an increase of 0.47 dB and 0.04 dB in the active-bias and off-state conditions, respectively. In the IM case, it showed a decrease of 0.82 dB and an increase of 0.2 dB in the active-bias and off-state conditions, respectively. Lastly, both the FM and IM voltage amplifiers exhibited no degradation in the diode-connection stress. It is interesting to observe that the voltage amplifier shows a relatively minor change in performance for the given stress time, compared with the TIA. This implies that the SiGe HBTs will present more robust operation if they are driven by input voltages rather than input currents.

Table 5 and Table 6 summarize the degradation characteristics of the circuits under investigation in terms of gain and bandwidth. From Table 5, the gain of the FM SiGe-HBT TIA under the active-bias stress was the most sensitive, whereas the diode-connection and the off-state stress cases do not lead to significant performance degradations. When it comes to the IM operation, SiGe-HBT TIA showed the least degradation under the diode connection, whereas the active bias and off state showed similar degradation to the FM counterparts. Investigations at the device and circuit levels provide insight into the degradation characteristics of SiGe HBTs in the three stress conditions and the impact of the degradation characteristics in circuit performance. Therefore, it can be expected that degradation at the system level can be predicted in advance, thereby reducing the stress-induced degradation of the circuit and analyzing for a potential lifetime improvement of the circuit. First, when the circuit is in a non-operational state, biasing it in active-bias conditions can lead to unwanted degradation. Second, if the device is not in use, it can be biased in the off state in FM. On the other hand, when IM is used, putting it in the off-state condition will degrade the device performance. Thus, it is safe for IM SiGe HBTs to be in the diode connection for better long-term reliability as long as power consumption is acceptable. As shown in Table 6, the degree of degradations is much less in a voltage amplifier configuration. Since this is partly due to the circuit configuration where the input signal does not see the variations in *r_π_*, a more in-depth analysis is necessary for different input signal networks even if a voltage is used. In short, these results can be utilized in an early-phase reliability analysis of SiGe-based circuits and systems. In addition, by understanding the different characteristics of FM and IM, better design and optimization can be conducted.

## 5. Summary

Investigations on SiGe-HBT reliability have been conducted on both device- and circuit-level operations. Degradation characteristics of a SiGe HBT were monitored in three different stress conditions: forward bias, diode connection, and off state. For each case, current gain, transconductance, and base-to-emitter resistance were extracted for forward- and inverse-mode operations. Based on the degradation results, performance changes in SiGe-HBT TIAs and voltage amplifiers have been investigated, using small-signal models and analyses. In terms of amplifier gain and bandwidth, the variations in amplifier performance have been compared and it shows a close relationship with device degradation characteristics. The findings of this work will be useful for various SiGe-HBT circuit and system applications.

## Figures and Tables

**Table 1:** Degradation of FM SiGe HBTs under each stress (3000 s).

Degradations in Parameter	Active Bias	Diode Connection	Off State
Average Δ*β* (%) [0.6 V ≤ V_BE_ ≤ 0.9 V]	−47.89	−21.15	+0.96
Average Δ*g_m_* (%) [0.6 V ≤ V_BE_ ≤ 0.9 V]	+11.97	+1.38	+0.46
Average Δ*r_π_* (%) [0.6 V ≤ V_BE_ ≤ 0.9 V]	−53.46	−22.16	+1.48
Δ*β* (%) [V_BE_ = 0.75 V]	−54.75	−22.23	+4.88
Δ*g_m_* (%) [V_BE_ = 0.75 V]	+6.8	+1.14	+0.47
Δ*r_π_* (%) [V_BE_ = 0.75 V]	−60.82	−23.14	+5.25

**Table 2:** Degradation of IM SiGe HBTs under each stress (3000 s).

Degradations in Parameter	Active Bias	Diode Connection	Off State
Average Δ*β* (%) [0.6 V ≤ V_BE_ ≤ 0.9 V]	−45.94	−1.11	−37.16
Average Δ*g_m_* (%) [0.6 V ≤ V_BE_ ≤ 0.9 V]	+5.17	+1.21	−8.52
Average Δ*r_π_* (%) [0.6 V ≤ V_BE_ ≤ 0.9 V]	−46.93	−2.27	−39.75
Δ*β* (%) [V_BE_ = 0.75 V]	−48.73	−0.30	−51.20
Δ*g_m_* (%) [V_BE_ = 0.75 V]	+15.70	+1.25	+3.52
Δ*r_π_* (%) [V_BE_ = 0.75 V]	−56.89	−1.70	−53.21

**Table 3:** Parameter values of FM SiGe HBTs used in the amplifier.

Device Stress Conditions		*g_m_* (mS)	*r_π_* (kΩ)	*V_A_* (V)
Active bias	Before stress	220	1.10	36.65
	After stress	99.9	1.16	34.09
Diode connection	Before stress	220	1.10	36.65
	After stress	169	1.11	36.21
Off state	Before stress	220	1.10	36.65
	After stress	232	1.09	36.52

**Table 4:** Parameter values of IM SiGe HBTs used in the amplifier.

Device Stress Conditions		*g_m_* (mS)	*r_π_* (kΩ)	*V_A_* (V)
Active bias	Before stress	60	1.50	1.72
	After stress	28.9	1.33	1.72
Diode connection	Before stress	60	1.50	1.72
	After stress	59.7	1.50	1.68
Off state	Before stress	60	1.50	1.72
	After stress	28.3	1.55	1.64

**Table 5:** Predicted changes in gain and bandwidth of a TIA.

Device Stress Conditions		Forward Mode	Inverse Mode
Active bias	ΔGain (dB)	−6.37	−7.38
	ΔBW (%)	+95.49	+88.5
Diode connection	ΔGain (dB)	- *	- *
	ΔBW (%)	- *	- *
Off state	ΔGain (dB)	+0.42	−6.22
	ΔBW (%)	−4.41	+73.44

*: No degradation observed.

**Table 6:** Predicted changes in gain and bandwidth of a voltage amplifier.

Device Stress Conditions		Forward Mode	Inverse Mode
Active bias	ΔGain (dB)	+0.47	−1.06
	ΔBW (%)	−0.007	+0.023
Diode connection	ΔGain (dB)	- *	- *
	ΔBW (%)	- *	- *
Off state	ΔGain (dB)	+0.04	+0.30
	ΔBW (%)	-	−0.007

*: No degradation observed.

## Data Availability

Dataset available on request from the authors.

## References

[1] Thomas Z., Josef B., Fred B., Pascal C., Michael C., Bjorn D., Marina D., Philippe F., Sebastien F., Christophe G. (2021). SiGe HBTs and BiCMOS Technology for Present and Future Millimeter-Wave Systems. IEEE J. Microw..

[2] Washio K. (2004). High-speed SiGe HBTs and their applications. Appl. Surf. Sci..

[3] Yu C., Yuan J.S., Shen J., Xiao E. (2006). Study of Electrical Stress Effect on SiGe HBT Low-Noise Amplifier Performance by Simulation. IEEE Trans. Device Mater. Rel..

[4] Zhang J., Guo Q., Guo H., Lu W., He C., Wang X., Li P., Liu M. (2016). Impact of Bias Conditions on Total Ionizing Dose Effects of 60Coγ in SiGe HBT. IEEE Trans. Nucl. Sci..

[5] Kim T., Ryu G., Lee J., Cho M.-K., Fleetwood D.M., Cressler J.D., Song I. (2024). Simple Modeling and Analysis of Total Ionizing Dose Effects on Radio-Frequency Low-Noise Amplifiers. Electronics.

[6] Wei J.-N., He C.-H., Li P., Li Y.-H., Guo H.-X. (2019). Impact of displacement damage on single event transient charge collection in SiGe HBTs. Nucl. Instrum. Methods Phys. Res. Sect. A Accel. Spectrometers Detect. Assoc. Equip..

[7] Pan X., Guo H., Lu C., Zhang H., Liu Y. (2023). The Inflection Point of Single Event Transient in SiGe HBT at a Cryogenic Temperature. Electronics.

[8] Adekoya M.A., Liu S., Wang C., Du X., Xing T., Wang X., Li H., Guo Y., Zhou J., Zhang X. (2024). Simulation and analysis of the single event transient characteristics of SiGe HBT at low-temperature environment. J. Instrum..

[9] Zhang Z., Guo G., Li F., Sun H., Chen Q., Zhao S., Liu J., Ouyang X. (2023). Effects of Different Factors on Single Event Effects Introduced by Heavy Ions in SiGe Heterojunction Bipolar Transistor: A TCAD Simulation. Electronics.

[10] Zhang J.-X., He C.-H., Guo H.-X., Tang D., Xiong C., Li P., Wang X. (2015). 3-D simulation study of single event effects of SiGe heterojunction bipolar transistor in extreme environment. Microelectron. Reliab..

[11] Marshall P.W., Carts M.A., Campbell A., McMorrow D., Buchner S., Stewart R., Randall B., Gilbert B., Reed R.A. (2000). Single event effects in circuit-hardened SiGe HBT logic at gigabit per second data rates. IEEE Trans. Nucl. Sci..

[12] Adekoya M.A., Liu S., Wang X., Xing T., Li H., Meng F., Du X., Li Z., Huang T. (2024). Simulation and analysis of inverse-mode operation of single event transient mechanisms on NPN-SiGe HBT. Phys. Scr..

[13] Song I., Cho M.-K., Oakley M.A., Ildefonso A., Ju I., Buchner S.P., McMorrow D., Paki P., Cressler J.D. (2017). On the Application of Inverse-Mode SiGe HBTs in RF Receivers for the Mitigation of Single-Event Transients. IEEE Trans. Nucl. Sci..

[14] Song I., Raghunathan U.S., Lourenco N.E., Fleetwood Z.E., Oakley M.A., Jung S., Cho M.-K., Roche N.J.-H., Khachatrian A., Warner J.H. (2016). An Investigation of the Use of Inverse-Mode SiGe HBTs as Switching Pairs for SET-Mitigated RF Mixers. IEEE Trans. Nucl. Sci..

[15] Cressler J.D. New Developments in SiGe HBT Reliability for RF Through mmW Circuits. Proceedings of the 2021 IEEE International Reliability Physics Symposium (IRPS).

[16] Phillips S.D., Moen K.A., Lourenco N.E., Cressler J.D. (2012). Single-Event Response of the SiGe HBT Operating in Inverse-Mode. IEEE Trans. Nucl. Sci..

[17] Wei J.-N., He C.-H., Li P., Li Y.-H., Guo H.-X. (2020). Impact of layout and profile optimization for inverse-mode SiGe HBT on SET and TID responses. Microelectron. Reliab..

[18] Song I., Cho M.-K., Lourenco N.E., Fleetwood Z.E., Jung S., Roche N.J.-H., Khachatrian A., Buchner S.P., McMorrow D., Paki P. (2017). The Use of Inverse-Mode SiGe HBTs as Active Gain Stages in Low-Noise Amplifiers for the Mitigation of Single-Event Transients. IEEE Trans. Nucl. Sci..

[19] Song I., Cardoso A.S., Ying H., Cho M.-K., Cressler J.D. (2018). Cryogenic Characterization of RF Low-Noise Amplifiers Utilizing Inverse-Mode SiGe HBTs for Extreme Environment Applications. IEEE Trans. Device Mater. Rel..

[20] Fischer G.G., Molina J., Tillack B. Comparative study of HBT ageing in a complementary SiGe:C BiCMOS technology. Proceedings of the 2013 IEEE Bipolar/BiCMOS Circuits and Technology Meeting (BCTM).

[21] Kamrani H., Jabs D., d’Alessandro V., Rinaldi N., Jacquet T., Maneux C., Zimmer T., Aufinger K., Jungemann C. (2017). Microscopic Hot-Carrier Degradation Modeling of SiGe HBTs Under Stress Conditions Close to the SOA Limit. IEEE Trans. Electron Devices.

[22] Ghodsi H., Kaatuzian H. (2019). Analysis and design of a SiGe-HBT based terahertz detector for imaging arrays applications. Microelectron. J..

[23] Fischer G.G. Analysis and modeling of the long-term ageing rate of SiGe HBTs under mixed-mode stress. Proceedings of the 2016 IEEE Bipolar/BiCMOS Circuits and Technology Meeting (BCTM).

[24] Alaeddine A., Kadi M., Daoud K. Performance and structure degradations of SiGe HBT after electromagnetic field stress. Proceedings of the 2011 International Reliability Physics Symposium.

[25] Orner B.A., Liu Q.Z., Rainey B., Stricker A., Geiss P., Gray P., Zierak M., Gordon M., Collins D., Ramachandran V. A 0.13 /spl mu/m BiCMOS technology featuring a 200/280 GHz (f/sub T//f/sub max/) SiGe HBT. Proceedings of the 2003 Bipolar/BiCMOS Circuits and Technology Meeting (IEEE Cat. No.03CH37440).

[26] Couret M., Fischer G., Lopez I.G., Matos M.D., Marc F., Maneux C. Impact of SiGe HBT hot-carrier degradation on the broadband amplifier output supply current. Proceedings of the ESSDERC 2019—49th European Solid-State Device Research Conference (ESSDERC).

[27] Xiong C., Li Y., Liu S., Tang D., Zhang J., He C. (2015). Hot carrier effect on a single SiGe HBT’s EMI response. Microelectron. Reliab..

[28] Chae M., Kim H. (2023). Time- and Temperature-dependent Degradation of p-GaN Gate HEMTs under Forward Gate Voltage Stress. J. Semicond. Technol. Sci..

[29] Mukherjee C., Marc F., Couret M., Fischer G.G., Jaoul M., Céli D., Aufinger K., Zimmer T., Maneux C. (2020). A physical and versatile aging compact model for hot carrier degradation in SiGe HBTs under dynamic operating conditions. Solid-State Electron..

[30] Yang Z., Guarin F., Hostetter E., Wang P.-C. Hot carrier reliability of high-speed SiGe HBT’s under accelerated collector-base avalanche bias. Proceedings of the 7th International Caribbean Conference on Devices, Circuits and Systems.

[31] Weimer C., Fischer G.G., Schröter M. (2024). Characterization, Analysis, and Modeling of Long-Term RF Reliability and Degradation of SiGe HBTs for High Power Density Applications. IEEE Trans. Device Mater. Rel..

[32] Lee C.-I., Lin Y.-T., Lin W.-C. (2016). Analysis of temperature dependence of linearity for SiGe HBTs in the avalanche region using Volterra series. Microelectron. Reliab..

[33] Lee C.-I., Lin Y.-T., Su B.-R., Lin W.-C. (2015). SiGe HBT Large-Signal Table-Based Model With the Avalanche Breakdown Effect Considered. IEEE Trans. Electron Devices.

[34] Rickelt M., Rein H.-M., Rose E. (2001). Influence of impact-ionization-induced instabilities on the maximum usable output voltage of Si-bipolar transistors. IEEE Trans. Electron Devices.

[35] Veenstra H., Hurkx G.A.M., Goor D., Brekelmans H., Long J.R. (2005). Analyses and design of bias circuits tolerating output voltages above BV/sub CEO/. IEEE J. Solid-State Circuits.

[36] Jacquet T., Sasso G., Chakravorty A., Rinaldi N., Aufinger K., Zimmer T., d’Alessandro V., Maneux C. (2015). Reliability of high-speed SiGe:C HBT under electrical stress close to the SOA limit. Microelectron. Reliab..

[37] Yu C., Xiao E., Yuan J.S. (2005). Voltage stress-induced hot carrier effects on SiGe HBT VCO. Microelectron. Reliab..

[38] Lee D.H., Jeong H.S., Kim Y.G., Kim M.H., Son K.S., Lim J.H., Song S.H., Kwon H.I. (2023). Quantitative Analysis of Channel Width Effects on Electrical Performance Degradation of Top-gate Self-aligned Coplanar IGZO Thin-film Transistors under Self-heating Stresses. J. Semicond. Technol. Sci..

[39] Lee C.-I., Li S.-C., Zhang S.-Y., Yang J.-H. (2023). Deep Neural Network RF Model for SiGe HBT in Breakdown Regime. IEEE Microw. Wirel. Technol. Lett..

[40] Alaeddine A., Kadi M., Daoud K., Mazari B. (2009). Effects of electromagnetic near-field stress on SiGe HBT’s reliability. Microelectron. Reliab..

[41] Mukherjee C., Jacquet T., Fischer G.G., Zimmer T., Maneux C. (2017). Hot-Carrier Degradation in SiGe HBTs: A Physical and Versatile Aging Compact Model. IEEE Trans. Electron Devices.

[42] Mukherjee C., Fischer G.G., Marc F., Couret M., Zimmer T., Maneux C. (2020). A unified aging compact model for hot carrier degradation under mixed-mode and reverse E-B stress in complementary SiGe HBTs. Solid-State Electron..

[43] Weimer C., Jin X., Fischer G.G., Schröter M. Characterization of Dynamic Large-Signal Operating Limits and Long-Term RF Reliability of SiGe HBTs. Proceedings of the 2022 IEEE BiCMOS and Compound Semiconductor Integrated Circuits and Technology Symposium (BCICTS).

[44] Cadence Virtuoso ADE Suite. https://www.cadence.com/en_US/home/tools/custom-ic-analog-rf-design/circuit-design/virtuoso-ade-suite.html.

